# Behavioral and Physiological Alterations in Angus Steers Grazing Endophyte-Infected Toxic Fescue during Late Fall

**DOI:** 10.3390/toxins15050343

**Published:** 2023-05-18

**Authors:** Ignacio M. Llada, Jeferson M. Lourenco, Mikayla M. Dycus, Jessica M. Carpenter, Garret Suen, Nicholas S. Hill, Nikolay M. Filipov

**Affiliations:** 1 Interdisciplinary Toxicology Program, University of Georgia, Athens, GA 30602, USA; ignacio.llada@uga.edu; 2 Department of Physiology and Pharmacology, University of Georgia, Athens, GA 30602, USA; jessica.carpenter@uga.edu; 3 Department of Animal and Dairy Science, University of Georgia, Athens, GA 30602, USA; jefao@uga.edu (J.M.L.); madison.dycus@uga.edu (M.M.D.); 4 Department of Bacteriology, University of Wisconsin, Madison, WI 53706, USA; gsuen@wisc.edu; 5 Department of Crop and Soil Sciences, College of Agriculture, University of Georgia, Athens, GA 30602, USA; nhill@uga.edu

**Keywords:** tall fescue, *Epichloë coenophiala*, fescue toxicosis, pasture behavior, thermoregulation

## Abstract

Fescue toxicosis is caused by grazing ergot alkaloid-producing endophyte (*Epichloë coenophiala*)-infected tall fescue (E+). Summer grazing of E+ leads to decreased productivity, associated impaired thermoregulation, and altered behavior. The goal of this study was to determine the role of E+ grazing-climate interaction on animal behavior and thermoregulation during late fall. Eighteen Angus steers were placed on nontoxic (NT), toxic (E+) and endophyte-free (E−) fescue pastures for 28 days. Physiological parameters, such as rectal temperature (RT), respiration rate (RR), ear and ankle surface temperature (ET, AT), and body weights, were measured. Skin surface temperature (SST) and animal activity were recorded continuously with temperature and behavioral activity sensors, respectively. Environmental conditions were collected using paddocks-placed data loggers. Across the trial, steers on E+ gained about 60% less weight than the other two groups. E+ steers also had higher RT than E− and NT, and lower SST than NT post-pasture placement. Importantly, animals grazing E+ spent more time lying, less time standing, and took more steps. These data suggest that late fall E+ grazing impairs core and surface temperature regulation and increases non-productive lying time, which may be partly responsible for the observed decreased weight gains.

## 1. Introduction

Tall fescue, *Lolium arundinaceum*, is a worldwide distributed, temperate-climate perennial grass [1]. In the United States, it is grazed extensively in beef production systems covering at least 14,000 million hectares, primarily in the Southeastern parts of the country [2]. Its ability to resist pests, withstand drought, easy establishment, and resistance to intensive grazing pressure are some of the attributes responsible for its wide adoption [3,4]. This widespread adoption occurred before the mutualistic relationship between the plant and an endophytic fungus, *Epichloë coenophiala* was discovered [5]. This endophyte produces secondary metabolites that are responsible for the agronomic attributes of the plant [3], but endophytic metabolites also include ergot alkaloids (EA). EA are toxic to the grazing animal and responsible for losses in livestock production [6] by causing the most notorious pasture-related pathology: fescue toxicosis. This disease affects 8.5 million beef cattle in the United States alone and costs the beef industry almost 2 billion in U.S. dollars annually [7]. Consequently, wild-type endophyte-infected tall fescue is referred to as ‘toxic fescue’ (E+) [8]. Numerous management measures have been attempted to mitigate the adverse effects of this intoxication [9]. Amongst them is the replacement of E+ tall fescue with fescue cultivars that are inoculated with non-alkaloid producing endophyte strains [10,11], referred to here as a non-toxic endophyte (NT). However, E+ tall fescue pastures still dominate, and their replacement is both costly and environmentally impactful [2].

The most well-known mechanism of action of EA is their ability to mimic biogenic amines such as serotonin, (nor)epinephrine, and dopamine [12,13]. Structural similarity with these molecules allows EA to interact with their respective receptors in different tissues [14,15]. For example, due to EA’s serotonergic and noradrenergic activity, EA can induce vasoconstriction and alter the normal blood flow to peripheral and central tissues [16,17]. This, among others, is responsible for impaired thermoregulation of affected animals, which is essential for maintaining core body temperatures within normal range. Reduced weight gain after E+ grazing is another well-known symptom of fescue toxicosis. Although it can occur at any time of the year, periods of higher ambient temperature and humidity can exacerbate weight loss [2,18] Therefore, clinical presentation, as well as disease severity, are dependent on environmental pressures [19,20].

One of the mechanisms through which animals, including ruminants, regulate their body temperature is behavioral adaptation [21,22]. Under thermoneutral conditions, beef cattle spend 95% of their time grazing, walking, ruminating, and resting [23]. Grazing behavior has a diurnal rhythm with a peak in the early morning, coinciding with sunrise, and another one in the late afternoon, coinciding with sunset, with the latter being the most intense [23,24]. Lying is a high-priority behavior and excessive lying times might reflect cattle welfare [25]. Environmental conditions can modify normal behavior patterns. For example, in a warm environment animals will seek shade, reduce feed intake, increase water intake, increase standing time, and decrease normal resting time [21]. Data on beef cattle behavior while grazing E+ fescue is very limited. Nonetheless, previous findings indicate that preference for shade and other production-affecting behaviors are exacerbated by consumption of E+, likely due to decreased heat tolerance [2]. This can translate into poor animal performance, and even death [26]. In contrast, during cold weather and short days, in order to maintain their core body temperature and avoid heat loss, cattle seek shelter from inclement weather [27,28] and spend more time lying down [29]. It is well known that in a colder environment, cattle retain heat and maintain core body temperature by peripheral vasoconstriction [30,31]; such vasoconstriction can be markedly exacerbated by the EA-containing E+ fescue, and, in extreme cases, cause gangrene and necrosis in the distal extremities, including the ears and the tail [20,32]. However, little is known about how the interaction between E+ grazing, thermoregulation, and cattle behavior during colder weather impacts animal performance, particularly weight gain. 

The welfare of production animals is not only important to preserve animal health, but also to increase their productive potential [33]. New technologies, such as wearable electronic monitoring devices for livestock, have recently received considerable attention [34,35]. These non-invasive devices allow continuous monitoring of the physiological [36] and/or behavioral parameters of the animals [35]. The information generated can be used to gain a better understanding of disease as well as to detect early signs for disease prevention and/or mitigation. However, within the context of fescue toxicosis, the application of such non-invasive devices is limited. 

Given the absence of information on behavior and other physiological parameters in late-fall tall fescue grazing beef, the objective of this study was to use behavior and temperature monitoring devices and evaluate the interaction between consumption of toxic (E+) fescue by beef stockers and environmental temperature during late fall in the Southeastern United States (Georgia). Our hypothesis was that late-fall toxic fescue grazing would modify steer behavior and thermoregulation in a way that would adversely impact weight gain and some related physiological parameters. The results from this study provide new insights on the role of the interaction between E+ grazing and climate on animal behavior and productivity.

## 2. Results

### 2.1. Environmental Temperature

For the duration of the study (1–29 November 2021), the average daily temperature (T, in °C) was 9.9 (range: 4.4 to 15.3) and the average temperature-humidity index (THI) was 50.3 (range: 41.1 to 57.6). For the six sampling dates these two averages were 12.8 and 53.5 (Day 0; Pre), 10.3 and 51.1 (Day 2), 11.5 and 51.4 (Day 7), 8.2 and 49.1 (Day 14), 11.3 and 54.0 (Day 21), and 8.6 and 49.5 (Day 28), respectively. Environmental conditions during this study were about 1 °C colder than the average November temperatures for the previous ten-year period; the corresponding THI was also lower (three units) than the past 10-year November average of 53.1. These results suggest that throughout the study, the conditions were late fall-like and steers were not exposed to an environment conducive to even mild heat stress. However, it was slightly colder than usual.

**Figure 1:**
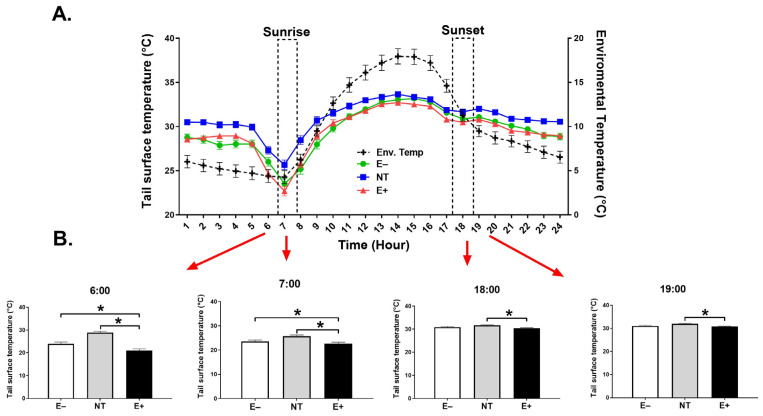
(**A**) Temperature (tail skin surface and environmental temperature, in °C)-treatment interaction during specific hours for the 28-day study period. (**B**) Tail Skin surface temperature (SST) 1 h before sunrise (6:00), the beginning of sunrise (7:00), beginning of sunset (18:00), and 1 h after sunset (19:00). (*) indicates a significant difference between treatment groups (*p* ≤ 0.05). Data are presented as mean ± SEM.

Within days, across the study, the average environmental T at 6:00, was 4.4 ± 0.7. At the beginning of the sunrise, it was 4.3 ± 0.7. One hour after sunrise, it was 9.5 ± 0.5. Early afternoon, it was 17.6 ± 0.8. At the beginning of sunset, and one hour after sunset, it was, respectively, 11.2 ± 0.6, and 9.5 ± 0.6 (Figure 1A).

### 2.2. Skin Surface Temperature (SST)

Throughout the grazing trial, the NT steers were able to maintain higher mean SST (*p* ≤ 0.001) than the E+ and E− group (30.9 ± 0.07, 29.5 ± 0.07, and 29.6 ± 0.07, respectively; Figure 1A, Table 1). This effect was only numerical for the first 14 days of the study (29.4 ± 0.11, 29.1 ± 0.11, 29.2 ± 0.11), after which it became highly significant (*p* ≤ 0.001) (32.3 ± 0.09, 29.9 ± 0.09, 29.9 ± 0.09).

At 6:00, the E+ group had significantly (*p* ≤ 0.05) lower SST (24.7 ± 0.3) compared to steers that grazed NT (27.3 ± 0.3) or E− tall fescue (26.0 ± 0.3). The same trend was observed at the sunrise hour, i.e., the E+ group had significantly (*p* ≤ 0.05) lower SST (22.7 ± 0.3) compared to NT (25.7 ± 0.3) or E− (23.5 ± 0.3) steers, with NT steers having the highest SST (*p* < 0.005; Figure 1B). At 9:00, the SST for the NT group was significantly (*p* < 0.001) higher than the other groups. Finally, at 14:00, 18:00 and 19:00, the SST was significantly (*p* < 0.05) higher in the NT steers. There were no significant differences observed between the E+ and E− groups at these two time points, but SST was numerically lower in the E+ group (Figure 1B, graphs for hours 9:00, and 14:00 are not shown because they follow the same trend as those for hours 18:00 and 19:00).

### 2.3. Behavioral Activity

The major significant differences in behavioral activity (standing time, lying time, number of steps) were observed during the light period (6:00–19:00); during darkness (20:00–05:00), there were no significant differences (*p* ≥ 0.2). Throughout the grazing trial, steers grazing E+ spent significantly (*p* ≤ 0.001) less time standing, more time lying down (*p* ≤ 0.001), and made significantly (*p* ≤ 0.01) or numerically more steps than, respectively, NT or E− grazing steers (Table 1). During the entire study, each E+ grazing steer spent, on average, 11.3 ± 0.39 days standing (10.1 ± 0.36 h/day), 15.7 ± 0.39 days lying down (13.9 ± 0.36 h/day) with a frequency of 10.8 ± 0.42 LB per day (Table 1) and made 1865.8 ± 8.79 steps/day (Table 1). Both the NT and E− steers spent more time standing and less time lying down (*p* ≤ 0.0001) than those in the E+ group. There were no significant differences (*p* ≥ 0.1) in the number of LB/day between groups. Additional behavioral parameter details for the 28-day trial are presented in Table 1. 

**Figure 2:**
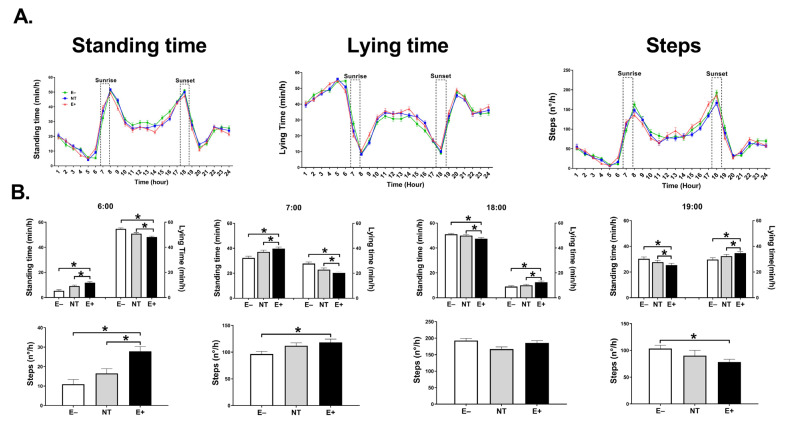
(**A**) Average 24-h behavioral activity throughout the trial. (**A**) Standing time (min/h)-left side, lying lime (min/h)-middle, and steps (n/h)-right side of Angus steers grazing E− (n = 6), NT (n = 6) and E+ (n = 6) tall fescue. (**B**) One-hour windows from (**A**); top panels: standing and lying time on specific hours of the study (6:00, 7:00, 18:00, and 19:00). Bottom panels: number of steps for the same hours. (*) indicates a significant difference between treatment groups (*p* ≤ 0.05) for these variables. Data are presented as mean ± SEM.

Notably, at 6:00 and at the beginning of sunrise, steers grazing E+ spent significantly (*p* ≤ 0.001) more time standing, less time lying down, and made more steps than the E− and NT group (Figure 2A). At 9:00, 14:00, 18:00, and 19:00, the most significant differences (*p* ≤ 0.001) between groups were observed in standing and lying times. During these hours steers grazing E+, spent significantly more time lying down, and less time standing than the E− and the NT groups (Figure 2B, graphs for hours 9:00, and 14:00 are not shown because they follow the same trend as those for hours 18:00 and 19:00). Regarding the number of steps within these hours, the only significant difference was observed at 19:00 when animals grazing E+ made a significantly (*p* < 0.005) and numerically (*p* < 0.1) fewer steps than E− and NT steers, respectively (Figure 2B). 

### 2.4. Endophyte and Total Plant Ergot Alkaloids

Mean endophyte presence in the E+ pastures was 78% and all plant tillers with endophyte tested positive for EA. Mean endophyte presence in the NT pastures was 92% with no EA production, and the E− paddocks had 0% endophyte presence. The overall total EA concentration in the whole tissue plant samples was 12.6 parts per billion (ppb) for the E+, while for NT, and E− pastures, the levels were below the detection limit for the test.

### 2.5. Nutritional Analyses and Forage Availability of Fescue Pastures

Forage quality in all pastures was high and there were no significant differences (*p* ≥ 0.1) between pastures in any of the parameters analyzed except for NDF values, which were significantly higher (*p* ≤ 0.05) in the NT tall fescue when compared to E−. Pasture nutritional analyses details are presented in Supplementary Table S2. Based on the formula described in [37,38], the apparent dry matter intake was calculated and it was not different between pastures (range: 2.7 to 2.9% BW). This, together with the amount of grass offered, established that ample forage was available in all the paddocks to support the 28 days of grazing the study encompassed. 

### 2.6. Animal Weight Gains and Physiological Parameters

**Figure 3:**
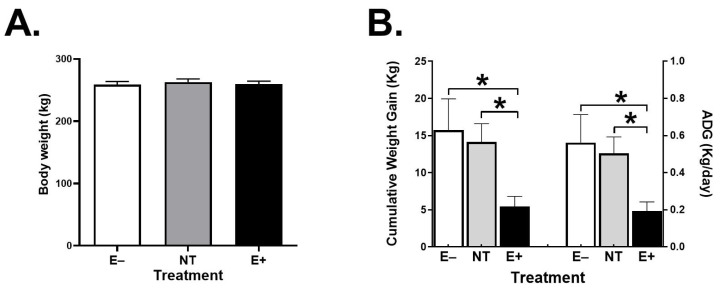
(**A**) Body weight (kg) of Angus steers before (pre) pasture placement, (**B**) Cumulative (kg) and Average daily weight gains (ADG; kg/day), after 28 days of grazing E− (n = 6), NT (n = 6) or E+ (n = 6) tall fescue. (*) indicates a significant difference between treatment groups (*p* ≤ 0.05). Data are presented as mean ± SEM.

Both cumulative and average daily weight gain (ADG) were significantly lower (*p* ≤ 0.01) in steers grazing E+ fescue at the end of the grazing trial (Figure 3). Overall rectal temperature (RT) was significantly higher (*p* ≤ 0.05) in the E+ steers (Table 1); this overall difference was most pronounced on days 14 and 21 of the grazing trial. No significant differences (*p* ≥ 0.2) were observed in respiration rate (RR) among treatments throughout the experiment (Table 1). However, in E+ steers RR was numerically higher on days 7, 14, 21, and 28. Regarding the spot ear temperature (ET), no significant differences (*p* ≤ 0.2) were observed between groups throughout the grazing trial (Table 1). Similarly, no significant differences (*p* ≥ 0.4) were observed in the spot ankle temperature (AT) (Table 1).

### 2.7. Ruminal pH, and Rumen and Fecal Volatile Fatty Acid (VFA) Concentrations

Throughout the grazing trial, animals grazing E+ had significantly lower (*p* ≤ 0.05) ruminal pH (7.15) than E− (7.27), and NT (7.30), while no significant difference was observed between the last two groups (*p* > 0.5). Post pasture placement, no significant differences (*p* > 0.2) were observed between groups for the total concentration of VFAs in the ruminal fluid, but total VFAs in the E+ steers were numerically higher (Supplementary Table S1). Within sampling days, only at the end of the study (day 28), the total rumen VFAs and butyrate concentrations were significantly higher (*p* ≤ 0.05) for the E+ compared to E− (Supplementary Figure S1A). When individual VFAs were analyzed across the trial, there were no significant differences in ruminal acetate, propionate, or butyrate between groups (*p* > 0.5), but they were numerically higher in the E+ steers (Supplementary Table S1). No significant differences (*p* > 0.4) were observed also in the acetate:propionate ratio (A:P), but it was numerically lower for the E+ group (Supplementary Table S1). 

In the fecal matter, the total VFAs and acetate concentrations were significantly (*p* < 0.01) higher in the E+ than in NT and numerically higher than E− post-pasture placement (Supplementary Table S1). This difference was most pronounced on days 7, 14 and 28 (Supplementary Figure S1B). Overall, no significant differences (*p* > 0.1) were observed in the propionate concentration between groups (Supplementary Table S2), but there were differences in this VFA on specific sampling days, i.e., on day 7 it was significantly higher (*p* ˂ 0.03) in E+ than NT and on day 14, fecal propionate was significantly higher (*p* ˂ 0.02) in E+ than E− (Supplementary Figure S1B). No differences (*p* > 0.5) were observed in the concentration of butyrate between groups nor in the A:P ratio overall or within sampling days (Supplementary Table S1 and Figure S1B). Additional details of the ruminal fluid and fecal matter VFAs dynamics can be found in Supplementary Figure S1A,B and in Supplementary Table S1.

## 3. Discussion

Two of the most frequently observed signs of fescue toxicosis are impaired thermoregulation [39] and low weight gain [3,40,41]. Although poor performance associated with the consumption of toxic fescue is a clinical sign that can be observed throughout the year [18], the inability for E+ grazing animals to regulate their body temperature is mostly documented during warm weather [42,43]. Little is known about the thermoregulatory capacity of steers consuming E+ fescue during mild or cold weather, at least in grazing trials. In the present fall grazing study, lowered weight gains and impaired thermoregulation were both observed. Overall, we found that steers grazing E+ during late fall had impaired core and surface temperature regulation. Moreover, alterations in SST were exacerbated during the coldest hours of the day, suggesting that ergot alkaloids or other active metabolites produced by the toxic endophyte alter the thermoregulatory ability in E+ grazing steers in an environment-dependent manner. Consequently, steers grazing E+ modified their behavior, as they spent more time lying down, less time standing, and took a greater number of steps daily. This interactive impact of toxic fescue grazing and climactic conditions on behavior could partly explain the poor weight gains of E+ steers when compared to the E− and NT groups. 

Steers grazing E+ during late fall had significantly lower (>60%) weight gains than steers grazing NT or E− tall fescue. These results are within the range reported by Paterson et al. [44], who compared 11 studies conducted across 10 U.S. states and found that animals consuming toxic fescue gained 30–100% less weight than animals consuming E−. In addition, several studies have demonstrated the superiority in weight gain of steers consuming NT vs. E+ [11,45].

Lower weight gains can be explained, in part, by a reduction of feed intake by animals consuming E+ [46], which may be linked to reduced palatability [47], depressed digestibility [48], and, notably, by the inability of animals to regulate their body temperatures, leading to alterations in their normal behavioral patterns [26,49]. Moreover, some studies have associated the poor performance of E+ steers with reduced absorption of VFAs, the main source of energy for cattle, in the rumen [50]. Although we did not measure dry matter intake, the overall quality of the forage, regardless of endophyte presence and status, was high. Notably, the concentration of total and individual VFAs in the rumen was numerically higher, while in the fecal matter, these concentrations were significantly higher in the E+ group. This is suggestive of alterations in the absorption process throughout the gastrointestinal tract. Interestingly, Welch et al. [51] found that inefficient steers had a greater concentration of total VFAs in their feces, compared to feed-efficient steers, indicating that higher fecal excretion of VFAs (most likely due to lower absorption) may be related to animal feed efficiency status. Thus, VFA concentrations along the gastrointestinal tract and their contribution to the pathogenesis of fescue toxicosis need to be investigated further and in greater detail.

We also found that animals consuming E+ fescue during fall had impaired core and surface temperature regulation, leading to behavioral adaptations that may be partly responsible for the poor performance observed during our study. For example, RT was significantly higher in the E+ group compared to animals consuming E− and NT, while SST was significantly lower when compared to the NT group and numerically lower than the E− group. The three groups of animals had values of RT slightly above the reference range for beef cattle (36.7–39.1 °C; average: 38.3 °C) [52]. The movement of the animals to the working area to collect data could explain this modest elevation, since walking activity alone is enough to raise body temperature in bulls, heifers, steers [53], and cows [54]. Although treatment differences in RT were not marked, its narrow physiological range on cattle might cause small oscillations to be significantly impactful on animal homeostasis as demonstrated in [55], where 0.1 °C increase in RT resulted in 4.8 more breaths per minute (bpm) in cattle.

Rhodes et al. [16], observed a reduction in peripheral and central blood flow in animals due to E+ consumption, which is likely due to the vasoconstricting effects of EA. During normal metabolism, there is constant heat production, which must be removed from the body to maintain core temperatures within the physiological range [31]. This heat, generated by different tissues, must be exchanged within the body, such that it reaches the skin vasculature where it is released into the environment through radiation, conduction, and convection [31,56]. The thermoregulatory center, located in the hypothalamus, is responsible for maintaining this core temperature through the activation of mechanisms necessary to retain or remove body heat (i.e., through peripheral (skin) vessel vasoconstriction or vasodilation) [31,57]. We infer that in our study, the peripheral vasoconstriction (SST) observed in the steers grazing E+ likely resulted in reduced heat dissipation produced during the normal metabolism and movement of the animals, which in turn increased core body temperature (RT). Moreover, the ingestion of EA can increase the core temperature by direct effect upon the thermoregulatory center in the brain [58], which can occur at a lower dosage compared to that necessary to mediate vasoconstriction [3]. Therefore, dysregulation of the thermoregulatory center could have also contributed to the hyperthermia observed in the E+ steers in our study.

We note that we did not observe significant differences in the respiration rates between groups. However, this rate was numerically higher on all sampling days in the E+ animals. Decreased tissue oxygenation due to the direct effects of alkaloids on receptors in lung tissue and platelets [8], which may be a possible explanation for increased RR, was likely not affected to a major extent in our study. Generally, the alteration of this parameter is frequently observed in animals grazing toxic fescue in warm environments with high THI [39,43] and is due to their inability to exchange body heat from the skin to the warm environment. The THI values observed in our study suggest that the animals were not exposed to heat stress. Moreover, during sampling days, we did not observe differences in the spot surface temperature at the base of the ear, nor at the surface of the ankle, which would allow us to explain a lower heat dissipation. These findings suggest that the vasoconstrictor effect generated by the consumption of E+ is dependent on the ambient temperature [39]. In this regard, the greatest differences in SST, which we monitored continuously, were observed at the coldest hours of the day (i.e., at 6:00 and 7:00), when the average ambient temperatures were 4.4 ± 0.7 and 4.3 ± 0.7 °C, respectively. During these hours, E+ grazing animals had significantly lower SST than NT or E− steers.

The environmental temperature at which animals do not spend additional energy to maintain their body temperature is known as the thermoneutral zone (TNZ) [56]. Below (lower critical temperature, LCT) or above (upper critical temperature, UCT) the TNZ, animals must increase heat production or heat loss to maintain their core temperature [59,60]. The point at which the LCT is reached can be variable and does not necessarily occur at a specific temperature. For cattle, Wagner et al. [59] and Ames [61] indicated that the LCT temperature is 7 °C, while Aiken et al. [17] suggest that a temperature of 5.4 ± 4.6 °C is below the LCT. Below this temperature, animals begin to feel cold and will activate involuntary physiological mechanisms to retain heat through mechanisms such as peripheral vasoconstriction. Therefore, we posit that during the coldest hours of the current study, the physiological vasoconstrictor effect is triggered to retain heat, and was exacerbated in the steers grazing E+ fescue. During the remaining daytime hours (09:00 to 19:00), when the average ambient temperature ranged between 9.5 ± 0.5 and 17.6 ± 0.8, animals in the NT group had significantly higher SST than the animals in E+ and E−, with no differences observed between the latter two groups. However, heat dissipation through the skin was numerically lower in E+. This is consistent with the observations by Aldrich et al. [42], where no differences in skin vaporization were observed between E+ and E− at thermoneutral temperatures.

We also found that animals grazing NT tall fescue was able to maintain a higher SST across the entire trial and had fewer steps while standing. This suggests that they were likely grazing and not walking and might be due to optimal thermoregulation. Because of that, the energy demand required to maintain the body temperature by NT steers within the TNZ might be lower and, consequently, more will be shifted into production. Additionally, likely higher skin vaporization under NT grazing conditions could be a promising advantage in warm environments where the animals could deal with potential heat stress. The utilization of different varieties of tall fescue inoculated with a non-toxic endophyte strain [9,10] has been a welcomed addition to technological breakthroughs that attempt to mitigate fescue toxicosis [8], but its adoption by farmers is limited due to a variety of factors, including cost. In addition to being effective in mitigating the clinical signs of the disease [9,11] and in improving plant persistence [10,12], the findings from this study point to another potential benefit of the NT technology. This needs to be validated in future experiments.

Another mechanism that animals use to cope with environmental temperatures outside the TNZ is through behavioral adaptations [21,22]. Unlike physiological regulation by the hypothalamus, this is voluntary. Under thermoneutral conditions, beef cattle spend 95% of their time grazing, walking, ruminating, and resting [23]. Grazing behavior has a diurnal rhythm with a peak in the early morning, coinciding with sunrise, and another in the late afternoon, coinciding with sunset, with the latter being the most intensive [23,24]. This grazing pattern was reinforced early on by Hughes and Reid [62], where they observed that the beginning and end of grazing coincided with dawn and dusk, respectively. This is the primary reason why we focused on six specific one-hour windows within the 24-h period in our study.

In this regard, during the entirety of our study, we observed that animals grazing E+ fescue did not exhibit gross aberrations from normal behavior (i.e., animals from all three groups were most active during sunrise and sunset). However, there were significant differences between groups with respect to the intensity of the activities performed. Overall, animals grazing E+ spent significantly more time lying down, less time standing and, when they were standing, exhibited a greater number of steps. On average, the animals grazing E+ spent 30 min/day more lying down and less standing in comparison with animals in the NT and E− groups. Lying is a high-priority behavior for cows. In fact, when access to feed or lying areas is restricted, lying time has been shown to have a higher priority over other behaviors, such as eating [63]. Therefore, variations in lying times might reflect cattle welfare [25,63]. Studies showed that dairy cattle with moderate to severe lameness increased on average 35–40 min/day the time that they spent lying when compared with healthy animals [64,65]. The time our animals spent lying down was similar. This late-fall behavioral pattern differs from that observed in steers consuming E+ fescue during summer where animals spend more time standing in shadows and spend less time lying, possibly in an attempt to increase their body surface area for more efficient heat removal [21,66]. In our late-fall grazing study, the increased lying time over standing time may be a mechanism to reduce body surface area and avoid heat loss. Normally, during cold weather, ungulates spend 40–50% of each day lying down [67]. In our study, all groups were within this range, but lying time was significantly higher in the E+ group, possibly due to a poor thermoregulatory capacity. In a nutshell, we suggest that the increase in lying time may have reduced the grazing time in steers grazing E+. In addition, since we did not observe differences in behavior activity during the hours of darkness, we contend that the lower activity detected during the day was not compensated during the night.

The intensity of these behavioral activities varied according to ambient temperature and the SST. During the coldest hour, when the lowest SST was recorded, animals grazing E+ spent more time standing and had a greater number of steps. In contrast, when the daily environmental temperature was higher between 09:00 and 19:00, the most significant differences in behavior were in lying and standing times (i.e., E+ steers spent more time lying down and less time standing). A possible explanation for this observation is that due to a likely vasoconstrictor effect of E+ consumption, less central body heat reaches the skin, thereby generating a cold sensation in the animal when the SST is lower. This may cause animals consuming E+ to be more active during cooler hours in an attempt to generate extra heat [21,22,68]. As the ambient temperature begins to rise, the animals spend more time lying down, perhaps to reduce the surface area exposed to the environment in order to retain heat. This may become a double-edged sword, since it may not only be harder to dissipate the heat afterward, but may also lead to less active grazing. 

Ergovaline (EV) accounts for 84–97% of the total ergot alkaloids in the fescue plant [69,70], which is why it has been considered the main pathological ergot alkaloid constituent of E+ tall fescue [71,72]. Therefore, different studies have tried to establish a threshold of EV and/or total ergot alkaloid intake to reduce the negative impact of this disease [73]. In our study, the total ergot alkaloids in the E+ pasture, compared to other reports [11,74,75] were low. This may suggest that: i) other non-ergot fungal metabolites could be involved in the pathophysiology of fescue toxicosis; or ii) even low ergot alkaloid levels in the fescue plant, when combined with late-fall colder temperatures, are sufficient to negatively impact animal behavior, thermoregulatory capacity, and productivity (weight gains).

## 4. Conclusions

Steers grazing E+ tall fescue during late fall displayed signs of fescue toxicosis, such as impaired thermoregulation and lower weight gain. The incorporation of wearable electronic monitoring devices (for behavior and temperature) in our study of fescue toxicosis allowed us to gain new insights into the role of the interaction between toxic fescue grazing and climate on animal behavior. Data presented here provide evidence that late fall E+ grazing contributes to production losses by altering animal thermoregulatory capacity, leading to counterproductive behavioral adaptations.

## 5. Materials and Methods

### 5.1. Animals, Treatments, and Experimental Design

The study was conducted in the fall of 2021 (1 November to 29 November), on pastures located at the University of Georgia’s J. Phil Campbell Natural Resources Conservation Center (Watkinsville, GA, USA). Post-weaning steers (n = 18; BW = 259.1 ± 2.72 kg) were blocked by weight and randomly assigned to 0.4 ha of vegetative fescue pastures that were sown in the fall of 2020 with tall fescue containing new, non-toxic endophyte (NT; Jesup MaxQ strain AR542; Max-Q, 3 paddocks, 2 steers per paddock, n = 6; 263.1 ± 4.91 kg); a toxic endophyte (E+; Jesup with wild-type endophyte, 3 paddocks, 2 steers per paddock, n = 6; 259 ± 5.05 kg); and endophyte-free fescue (E−; 3 paddocks, 2 steers per paddock n = 6; 258.9 ± 5.04 kg). The steers were kept on pasture for 28 days. Different parameters such as respiration rate (RR), rectal temperature (RT), surface temperature on the base of the ear (ET), and ankle temperature (AT) were collected before (pre) and at 2, 7, 14, 21 and 28 days after pasture allocation. On those days, fecal matter and rumen contents were collected. Steer live body weights were recorded before (pre) and at 14 and 28 days after pasture placement using a digital scale. Fescue plants were collected on the first and the last day of the trial for different analyses, as described below. All samples and clinical parameters were collected between 08:00 and 12:00.

On day 1 of the experiment (pre) all animals were equipped with wireless temperature sensors (iButton data loggers, Maxim integrated, San Jose, CA, USA) affixed to the base of the tail with a self-adhesive estrotec sticker (Rockway Inc., Spring Valley, WI, USA) to measure the surface temperature of the skin (SST) continuously. Animals were also fitted with Icetag activity sensors (IceRobotics, Edinburgh, Scotland) on their right hind limb to record behavior. Environmental conditions (relative humidity and temperature) were measured using six data loggers (HOBO^®^ H8 Pro, Onset Computer, Bourne, MA, USA) placed in the paddocks where animals grazed and under shed houses used to protect the animals from inclement weather.

### 5.2. Sample Collection and Processing

Respiration rates were monitored by counting full flank breathing movements for 60 s, twice per animal, and by calculating the mean, similar to [43,76]. Rectal temperature was taken with a DeltaTrak digital thermometer (Pleasanton, CA, USA) after obtaining a stable reading for 15 s. The surface temperature was taken at the base of the right ear and right ankle of each animal using a Fluke 59 MAX+ IR Thermometer (Melrose, MA, USA). All temperatures were recorded in degrees Celsius (°C).

Fresh fecal samples were collected by hand using fresh gloves for each collection, placed in 50 mL conical centrifuge tubes, and stored on ice. Samples of ruminal contents were collected using an oro-ruminal probe, which was washed between animals as in [77]. To eliminate contamination with saliva, the first collection from each animal was discarded and the second was retained for future analysis. Approximately 50 mL of ruminal contents were filtered using 4 layers of autoclaved cheesecloth, which allowed ruminal liquid and solids separation. A portion of the rumen liquid was quickly placed in sterile cryovial tubes, and kept on dry ice; the remainder of the liquid was used for pH measurement using an Ecoscan pH150 portable pH-meter (Vernon Hills, IL, USA). Upon arrival at the laboratory, fecal and rumen samples were stored at −80 °C.

#### 5.2.1. Detection of Endophyte and Total Plant Ergot Alkaloids Analysis

Sampling was performed by selecting a tiller from 100 locations within the pastures, cutting the tiller at the soil surface, and transporting the samples to the laboratory. Endophyte presence was analyzed from a 3-mm cross-section of the stem base of each tiller using a commercial immunoblot test kit (Agrinostics Ltd., Co., Watkinsville, GA, USA, Cat. # ENDO797-3). In addition, total plant ergot alkaloid content was determined by a second tiller cross section using a commercial ELISA test kit (Agrinostics Ltd., Cat. # ENDO899-96p), with a limit of detection (LOD) of 1 ppb, as in [75]. 

#### 5.2.2. Nutritional Analyses and Forage Availability of Fescue Pastures

Pasture samples were collected from each individual paddock on the first day of the study and sent to the University of Georgia’s Agricultural and Environmental services Laboratories. All samples were analyzed by near-infrared spectroscopy (NIRS) using a FOSS NIRSystems model 6500 scanning monochromator (FOSS North America, Eden Prairie, MN, USA) as in [78,79]. The attributes or quality parameters measured in the pastures were: crude protein (CP), fat, inorganic matter (Ash), Neutral Detergent Fiber (NDF), Acid Detergent Fiber (ADF), Total Digestible Nutrients (TDN), lignin, NFC (non-structural carbohydrates), water-soluble carbohydrate (WSC), ethanol soluble carbohydrate (ESC), starch, Neutral Detergent Fiber Digestibility (dNDF48), Digestible Dry Matter (DDM48).

Forage availability was determined on each paddock using a pasture meter at the beginning of the study. In order to establish if the quantity of grass provided to the animals was enough to carry out the 28 days of study, we estimated the percentage of dry matter intake (DMI) as a percentage of body weight (BW). Since the neutral detergent fiber (NDF) has been used as an indicator of forage intake because it takes into account all fiber components (lignin, cellulose, and hemicellulose) of the plant [37], we used the equation DMI (%BW) = 120/NDF (%DM) as in [37,38] to estimate the DMI for the animals.

### 5.3. Data Loggers

#### 5.3.1. IceTag^®^ Leg Sensor

Behavioral data were recorded using an electronic activity monitor as in [80,81]. Each steer was fitted with an IceTag^®^ (IceRobotics, Edinburgh, Scotland) on its right hind limb. Prior to placement, these devices were activated using an IceReader wireless download device (IceReader, IceRobotics, Edinburgh, Scotland) along with the IceManager software (IceManager, IceRobotics, Edinburgh, Scotland). They were programmed to collect data every 15 min over the 28 days of the experiment. These monitors use tri-axial accelerometers to capture very detailed information about the animal’s movements and behavior. The device collects data on the lying bout (LB), defined as the period between the sensor switching from vertical to horizontal and then back to vertical. The lying time is recorded when the sensor is in the horizontal position, the standing time when the sensor is vertical, and finally, the step count estimates the number of times the animal lifts its legs according to the amount of force used [82]. After 28 days, the data were extracted and downloaded using the same software and IceReader wireless download device used for activation. The files provided by this software were used to calculate the standing and lying time in minutes/hour, a number of steps/hours, and the number of LB/day. We took into account those days in which the animals had the devices collecting data for 24 h. In that sense, the first and last days of the study were not included. Based on previous studies, LB ≤ 2 min was discarded [83,84]. 

#### 5.3.2. iButton Temperature Logger

iButton data loggers (model DS1922L) were used to measure skin surface temperature. They are small (16.25 mm × 5.89 mm) autonomous systems that measure temperature and record the result in a protected memory section. The devices were activated using the OneWireViewer software (Version 1.6) and programmed to collect data every 5 min. At the end of the 28-day trial, the data were extracted and downloaded using the same software. As with the devices to record behavioral activity, only the days with full 24-h data were used for the data analysis.

#### 5.3.3. Environmental Temperature Data Loggers

These loggers were used to measure the environmental conditions, such as relative humidity (RH) and temperature (T). The type used was HOBO^®^ H8 Pro. Each HOBO Pro Series logger has an internal temperature sensor. Before starting operations, these devices were activated using a BoxCar Pro 4 software. The six data loggers used were configured to collect data every 10 min. After 28 days, the data were extracted and downloaded with the same software. After performing two-way analyses of variance, to determine if the location on the different paddocks was a factor, no significant differences were observed between the loggers (*p* > 0.5), and the data from the six devices are combined. The temperature-humidity index (THI) was calculated using the equation THI= 1.8 × T − ($\frac{100\;-\;\mathrm{RH}}{100}$) × (T − 14.3) − 32 as in [85], where T is the ambient temperature in degrees centigrade and RH is the percent relative humidity. Moreover, using weather information providing by Georgia Automated Environmental Monitoring Network of the University of Georgia, we calculated the average daily temperature and THI for the month of November for the last ten years (2010–2020). These data were collected from a station located at J. Phil Campbell in Watkinsville, GA, USA.

### 5.4. Volatile Fatty Acid Analysis

Ruminal and fecal samples were analyzed according to the procedure described in Lourenco et al. [77]. Ruminal samples were centrifuged for 10 min at 10,000× *g*. One milliliter of the ruminal fluid supernatant was mixed with a 25% (*w/v*) metaphosphoric acid solution. The samples were frozen overnight, centrifuged, and the supernatant was mixed with ethyl acetate in a 2:1 ratio of ethyl acetate to the supernatant. Next, 0.5 mL of the top portion was transferred to screw-thread vials for analysis of volatile fatty acids (VFAs) in a Shimadzu GC-2010 Plus gas chromatograph (Shimadzu Corporation, Kyoto, Japan) equipped with a flame ionization detector and a capillary column (Zebron ZB-FFAP; 30 m × 0.32 mm × 0.25 μm; Phenomenex Inc., Torrance, CA, USA). The fecal samples (1 g) were processed in the same way with the only difference being that they had to be solubilized in distilled water (3 mL) prior to processing. 

### 5.5. Statistical Analysis

Statistical analyses were performed with Sigma Plot, v12.5 (Systat Software, Inc., San Jose, CA, USA). For the weight gains, RR, RT, ET, and AT we used a two-way analysis of variance, with days of sampling and fescue treatment set as the two independent variables. If significant (*p* ≤ 0.05) main effects or interactions were observed, the Holm-Sidak post hoc analysis was applied to separate significant differences. To analyze the behavioral data, and the skin surface temperature data (iButton data logger) we used a three-way analysis of variance, using treatment, day, and hour within a day as independent variables, and the behavioral activity or skin surface temperature as a dependent variable, respectively. If significant (*p* ≤ 0.05) differences were observed between treatment, day, hour, or interactions (treatment by day or treatment by hour) two-way analyses of variance were performed. After initial analyses, the most significant differences observed in SST and behavioral activity were by treatment-hour interaction. In order to analyze in depth those differences and the interaction with the environmental temperature (Env.T) we further divided the 24-h days into six one-hour windows: one hour before sunrise (6:00), beginning of sunrise (7:00), one hour after sunrise (9:00), early afternoon (14:00), beginning of sunset (18:00), and one hour after sunset (19:00). For the nutritional analyses of the pastures, we averaged the individual quality parameters for each treatment pen, and we performed one-way analysis of variance using treatment as independent variable and the quality parameters as dependent variables. Graphs were generated with GraphPad Prism 5 (La Jolla, CA, USA).

## Figures and Tables

**Table 1:** Average physiological and behavioral activity parameters post-pasture placement for the whole study period.

	Treatment			
	E+	E−	NT	*p*-Value
**Physiological parameters**				
Rectal temperature (°C)	39.5 ^b^ ± 0.07	39.3 ^a^ ± 0.06	39.3 ^a^ + 0.09	0.05
Skin surface temperature (°C)	29.5 ^b^ ± 0.07	29.6 ^b^ ± 0.07	30.9 ^a^ ± 0.07	0.001
Ear surface temperature (°C)	27.9 ± 1.23	25.2 ± 0.61	26.8 ± 0.63	0.2
Ankle surface temperature (°C)	20.03 ± 0.62	20.3 ± 0.59	20.5 ± 039	0.4
Respiration rate (BPM; breath per minute)	40.5 ± 1.70	38.01 ± 2.69	38.4 ± 2.20	0.7
**Behavioral activity**				
Standing time (h/day/animal)	10.1 ^b^ ± 0.36	10.6 ^a^ ± 0.34	10.5 ^a^ ± 0.26	0.0001
Laying time (h/day/animal)	13.9 ^b^ ± 0.36	13.4 ^a^ ± 0.34	13.5 ^a^ ± 0.26	0.0001
Steps (n/day)	1865.8 ^b^ ± 8.79	1864.4 ^ab^ ± 8.18	1771.1 ^a^ ± 8.38	0.01
Lying bouts (n/day)	10.8 ± 0.42	11.7 ± 0.41	10.5 ± 0.42	0.1

Mean values with a superscript in common do not differ with a level of α = 0.05 overall comparisons.

## Data Availability

Not applicable.

## Supplementary Materials

The following supporting information can be downloaded at: https://www.mdpi.com/article/10.3390/toxins15050343/s1, The supplementary materials file consists of: (i) Supplementary Figure S1: Dynamic of Volatile Fatty Acid within sampling days of Angus steers after 28 days of grazing E−, NT or E+ tall fescue in ruminal fluid, and fecal matter; (ii) Table S1: Ruminal and fecal volatile fatty acids concentration of steers grazing E+, E−, and NT tall fescue throughout the 28-days of the study period; (iii) Table S2, Nutritional analyses of E+, E−, and infected NT tall fescue pastures.
