# Supplementary material for: Behavioral and Physiological Alterations in Angus Steers Grazing Endophyte-Infected Toxic Fescue during Late Fall
Source: Toxins (Basel). 2023 May 18;15(5):343. doi: 10.3390/toxins15050343 (PMC10222549; doi:10.3390/toxins15050343)
Supplement: Supplementary file 1 [file toxins-15-00343-s001.zip › toxins-2390506-supplementary.pdf]

toxins

MDPI

Supplementary Materials: Behavioral and Physiological Alterations in Angus Steers Grazing Endophyte-Infected Toxic Fescue during Late Fall

Ignacio M. Llada, Jeferson M. Lourenco, Mikayla M. Dycus, Jessica M. Carpenter, Garret Suen, Nicholas S. Hill and Nikolay M. Filipov

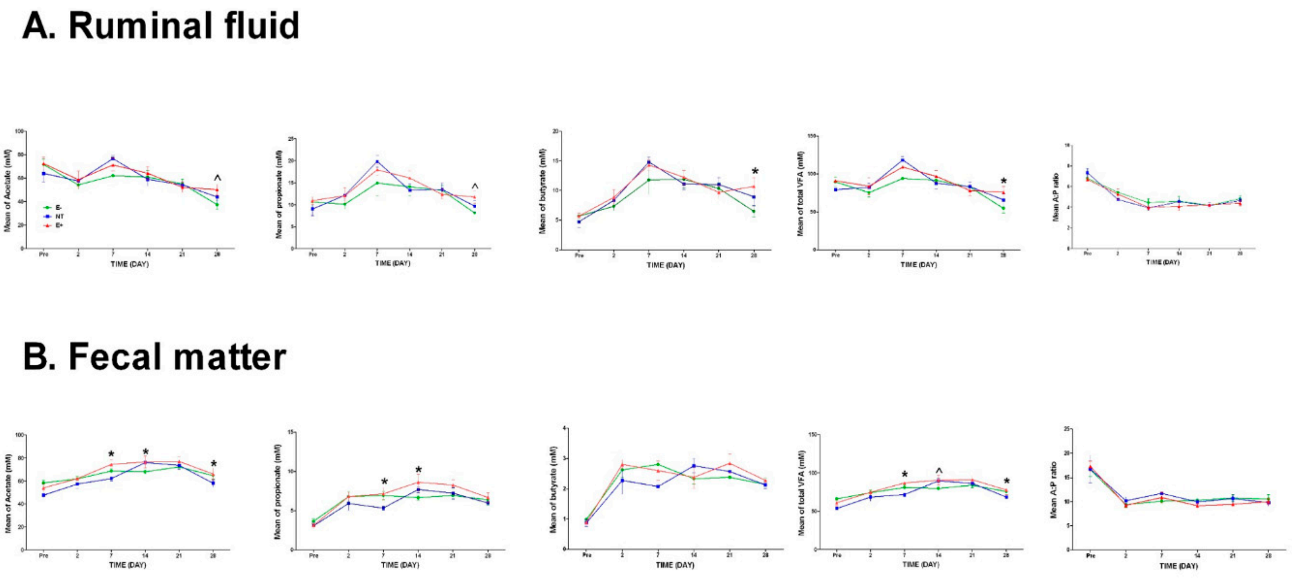

Figure S1: Dynamic of Volatile Fatty Acid (VFA) within sampling days of Angus steers after 28 days of grazing E- (n = 6), NT (n = 6) or E+ (n = 6) tall fescue in A) ruminal fluid, and B) fecal matter. (*) indicates a significant difference ($P \leq 0.05$), while (^) indicate trends ($P \geq 0.05$, $P < 0.1$) between E+ and other treatments. Data are presented as mean ± SEM.

Table S1. Ruminal and fecal volatile fatty acids (VFA) concentration of steers grazing endophyte-infected (E+), endophyte-free (E−), and nontoxic endophyte-infected (NT) tall fescue throughout the 28-days of study period.

**Treatment**	**E+**		**E-**		**NT**		**p-value**	
	Ruminal fluid	Fecal matter	Ruminal fluid	Fecal matter	Ruminal fluid	Fecal matter	Ruminal fluid	Fecal matter
**VFA (mM)**								
Acetate	61.5 ± 3.02	68.6^{a} ± 1.52	56.6 ± 3.02	65.6^{ab} ± 1.54	58.8 ± 3.24	62.2^{b} ± 1.57	0.56	0.03
Propionate	13.5 ± 0.89	6.82 ± 0.33	11.9 ± 0.89	6.22 ± 0.33	12.7 ± 1.02	5.97 ± 0.33	0.5	0.16
Butyrate	10.2 ± 0.73	2.32 ± 0.13	8.92 ± 0.73	2.21 ± 0.12	9.62 ± 0.77	2.11 ± 0.13	0.46	0.51
A:P ratio	4.77 ± 0.15	11.1 ± 0.45	5.04 ± 0.15	11.3 ± 0.44	4.94 ± 0.16	11.5 ± 0.46	0.47	0.74
Total VFA	88.7 ± 4.8	80.5^{a} ± 1.98	81.2 ± 4.8	76.6^{ab} ± 1.94	85.1 ± 5.02	72.7^{b} ± 2.03	0.53	0.04

Mean values with a superscript in common do not differ with a level of α=0.05 over all comparisons.

Table S2. Nutritional analyses of endophyte-infected (E+), endophyte-free (E−), and nontoxic endophyte-infected (NT) tall fescue pastures.

Item	E+	E-	NT	p-value
DM%				
CP	16.1 ± 0.7	17.3 ± 0.4	16.7 ± 0.7	0.55
Fat	2.86 ± 0.02	2.86 ± 0.04	2.81 ± 0.07	0.85
ASH	9.93 ± 0.2	9.88 ± 0.7	9.91 ± 0.5	0.99
NDF	41.7 ± 0.4[ab]	40.9 ± 0.4[a]	43.2 ± 0.7[b]	0.05
ADF	25.4 ± 0.3	24.7 ± 0.2	25.6 ± 0.5	0.34
TDN	68.5 ± 0.4	68.4 ± 0.7	67.4 ± 0.5	0.48
Lignin	2.32 ± 0.1	2.46 ± 0.02	2.42 ± 0.1	0.37
NFC	32.3 ± 0.6	32.6 ± 1.1	30.4 ± 0.5	0.22
WSC	16.6 ± 0.3	16.4 ± 0.6	15.1 ± 0.2	0.1
ESC	14.3 ± 0.3	14.2 ± 0.6	13.1 ± 0.1	0.12
Starch	2.34 ± 0.13	2.61 ± 0.1	2.41 ± 0.14	0.32
dNDF48	32.3 ± 0.6	31.3 ± 0.3	32.4 ± 0.2	0.22
DDM48	83.5 ± 1.1	83.4 ± 0.5	82.3 ± 0.6	0.52

Mean values with a superscript in common do not differ with a level of α=0.05 over all comparisons.

Crude protein (CP), inorganic matter (Ash), Neutral Detergent Fiber (NDF), Acid Detergent Fiber (ADF), Total Digestible Nutrients (TDN), NFC (non-structural carbohydrates), water soluble carbohydrate (WSC), ethanol soluble carbohydrate (ESC), Neutral Detergent Fiber Digestibility (dNDF48), Digestible Dry Matter (DDM48)
